# Injury incidence and balance in rugby players

**DOI:** 10.12669/pjms.306.5648

**Published:** 2014

**Authors:** Jaco Ras M, Threethambal Puckree

**Affiliations:** 1 Jaco Ras M. Physiotherapy, Sharks Rugby Academy and University of KwaZulu Natal,; 2 Threethambal Puckree, PhD, Durban University of Technology, Durban-4001, South Africa.

**Keywords:** Rugby injury incidence, Stability index, Limits of stability

## Abstract

***Objective*** *:* This study determined and correlated injury incidence and balance in rugby players.

***Methods:*** A prospective survey with balance testing was conducted on first year rugby academy players (N= 114). Injury incidence, static and dynamic balance were tested pre and post-season using a Biosway portable balance system. The data was analysed using paired and independent samples t-tests at p<0.05, Odds ratios, and Spearman’s correlation coefficients.

***Results:*** 75.50% participated, 71.40% were 18 years old, and 71.40% were White. Injury was sustained by 83% of players with the knee (25%) most commonly injured. Injury incidence was 1.52 per player with an injury rate of 5.95 injuries per 1000 match playing hours. The Stability Index increased significantly (p=0.03) by 15% in the medial/lateral direction post-season compared to pre-season. Significant differences in post-test anterior posterior and overall static and front and front right dynamic stability between injured and uninjured players were noted. Risk factors for injury included the scrum-half (14.80%) playing position, injuries in the 2nd half of the match (57%), and during contact (67%).

***Conclusion*** *: *Injury incidence was related to static and dynamic balance in forward right direction only.

## INTRODUCTION

Rugby is a popular contact sport with a higher risk of injury than other sports.^1^ It is characterized by high loading and the unique postural stances that predispose players to injury, similar to other sports with similar movements. Rugby players tend to adopt an unbalanced posture posteriorly resulting in difficulty in controlling foot stability resulting in a high prevalence of lower limb specifically ankle injuries.^2^^,^^3^ The control of static and dynamic balance requires a complex interplay between proprioceptive, vestibular and visual factors. Body load data revealed that high levels of gravitational force are sustained in tackling and scrum tasks.^4^ In South Africa, more publications on rugby related injuries are being published and a growing body of literature has been noted globally.^5^^,^^6^

Injury has been related to the nature of the sport, player position, and level of play.^2^^,^^4^ Injuries involving the shoulder, knee, and ankle caused significant absence from play in forwards, compared to the back-line players whose absence from play was attributed to injuries in the shoulder, hamstrings, and knees.^6^ The increasing emphasis on strengthening the core muscles not only to improve performance but also to reduce injuries has been welcomed by individuals participating in sports.^7^ Although coaching and training have incorporated the principles of strength, flexibility, specificity, intensity and duration into consideration, little attention has been paid to balance and stability testing or training. This study looked at whether any correlation exists between injury incidence and balance in rugby players.

## METHODS

A cross sectional survey was used to determine injury incidence in the first year members (N=101) of a rugby academy. The rugby academy prepares players to participate in a professional team. As players progress they become eligible for selection in the junior or professional team. Outstanding players in the professional team are selected to play in the National team. Balance was tested pre and post-season to enable correlation with injury incidence. 

Pre-season, after completing an informed consent and a demographic questionnaire, static and dynamic balance were tested using a calibrated portable Biosway balance device.^8^ For the postural stability test, the standard deviation of the stability index, reflected the sway index. This index measured static balance according to normative data from the Biosway balance system clinical test of sensory integration and balance (CTSIB). The normative sway index range is 0.21-0.48.8Good balance is set at ≤0.48. The sway index has an indirect relationship to static balance, therefore the less the sway index, the better the static balance or postural control.8The players were tested in bipedal stance, eyes open and on a firm surface.

For dynamic balance testing the limits of stability (LOS) was monitored by how accurately a player could move the display cursor to a target 10° from a platform zero position and back again. A higher score was obtained when the trajectory to the target and back was more direct. Low scores indicated poor neuromuscular control.^8^ The limits of stability results were categorized as good (LOS≥65%) or poor (LOS<65%) balance. The retest was performed after 60 seconds to improve reliability of the data. The same researcher conducted the balance testing pre-season and post-season and both tests was performed three times and an average calculated. Data were stored on the system logger and transferred to a computer for analysis.

After the test for normality of distribution, the data was analysed using paired and independent samples t-tests, Pearsons correlations and calculation of Odds Ratios at a p<0.05. Injury incidence, was expressed as injury rate calculated as the number of injuries divided by the match playing hours, and multiplied by a ratio of 1000 hours divided by match playing hours. This gave the injury incidence per 1000 match playing hours. The relative injury risk ratio was calculated by multiplying the injury severity (days absent) with the injury rate.

## RESULTS

The participation rate was 100% (n= 101) pre=season and 75.50 % (n=77) post season. The majority of the players were 18 (71.40%) and 19 years old (20.80%), with a range between 17 to 20 years (X± SD=18.32 ± 0.60 years). White players made up the majority (71.4%) of the cohort followed by Blacks (26%) and Coloureds (2.60%). 

 In the 2012 season, 83% of players were injured sustaining 117 injuries. Seventeen percent of the players reported no injuries. Equal proportions of the remaining players sustained either one (27 % of all injuries) or more than one injury (73% of all injuries). Injury rate was 1.52 ± 1.23 per player. The incidence of injuries by match playing hours was 5.95 per 1000 match playing hours. The mean number of matches played per player was 17.39 ± 7.30 (range = 4-40) matches and mean number of match hours per player was 21.89 ± 10.16 (range = 5-53). 

Injuries in the lower limb, upper limb and head and face accounted for 55%, 22% and 7% respectively of all injuries. The knee (25%), ankle (21%) and shoulder (15%) were most commonly injured. Lower back injuries made up 3% of the injuries. Table-I.

The scrum-halfs reported the most injuries (16%) with the least sustained by players in the loose-head lock (2%). The backs reported more injuries (57%), compared to the forwards (43%).The majority of the injuries occurred during contact (67%), during matches (66%), mainly in the 2nd half (57%) and due to trauma (62%). The majority of the injuries (81%) resulted in four or more days lost from play. About one fifth of injuries were mild (loss of 4-7 days), 37%were moderate (loss of 8-28 days) and 25% severe (loss of greater than 28 days).

***Sway index:*** The sway index reflects postural stability. It suggests ‘good’ balance if it is ≤0.48 degrees, but considered ‘poor’ if >0.48 degrees. As shown in Table-II, the odds of injured players with poor balance in the sagittal plane (anterior posterior sway) pre-season and in the frontal plan (mediolateral sway) post-season, sustaining injuries was high.

The mean medial/lateral SI score suggests good postural stability pre- and post-test. Mean post-test did not improve except for anterior posterior and overall SI in injured players. A significant difference between injured and uninjured post-test AP and overall SI was observed. No significant correlation between injury and pre or post-test stability index was noted.

***Limits of Stability (LOS): ***The LOS is an indicator of dynamic stability in the standing individual. The LOS is a measure of ‘good’ balance if ≥ 65%, but considered ‘poor’ if< 65%. In the pre-test, 23% of the injured and uninjured participants had good overall LOS compared to 39% in both groups post-test. Players with poor control in forward, backward, right, backward right and backward left were more at risk for injury pre-test compared to those whose control in the forward left, left, right and forward direction in the post-test was compromised. 

***Relationship between injury and dynamic balance:*** Dynamic balance in injured players improved only in movement to the back right and worsened to the front right. In uninjured players balance improved when moving to all directions except for back left, back right and front right post-test, significant differences between injured and uninjured players were noted in movements to the right and front right. 

As shown in Table-III, mean directional control (LOS) in only two directions was good. In the post-test, good scores were recorded in five of the eight directions of movement. A significant difference in dynamic balance to the front and right was observed between injured and uninjured players post-test. A significant correlation between forward right directional control and injury incidence was noted (R= - 0.218; p<0.05). 

## DISCUSSION

This study focused on first year players enrolled in a rugby academy which allows selection to play professionally and in the national team. The participation rate was excellent and supported by the managers and coaches. The demographic profile of the participants reflects the historic development in the game. It was a game in which only White people participated and since 1994 other race groups have been included.

A lower injury incidence than that reported in other rugby studies was noted.^5^^,^^9^ Haseler et al*.*, reported an injury rate of 24 injuries per 1000 match playing hours in amateur and youth rugby players in England.^9^ The injury definition was similar to that used in this study, but the method of collecting data was through the coach or first aider instead of the player. In addition, the observers who collected the data had a pre-season educational session to improve data accuracy. The difference in injury rate between this study and other studies could be attributed to the difference in data collection methods, or definitions of injury, as identified by the IRB consensus statement drawn up in a study by Fuller et al.^10^ This study used the IRB consensus statement definition for injury. Garraway and Macleod reported 14% of injuries in categories with more than one injury compared to the 73% in the current study.^11^ The differences could be due to these authors ascribing their injuries only to those that were severe enough to receive medical attention. Injury records from the hospital, attending physician or investigator were used in the analysis.

Lower limb injuries were the most common (55%) similar to Barthgate et al. who reported 52% of injuries in international level male rugby players.^12^ Haseler et al*. *reported that injuries commonly involved the knee, ankle and shoulder in junior male rugby players similar to our findings.^9^ McGuine et al. reported that previous sprains predisposed the ankle to new injuries due to disturbed balance.^13^ With professionalism and greater incentives to play associated with more training time, players are more likely to play with chronic or recurring injuries to keep these incentives.^12^

**Table-I:** Numbers of injured/uninjured players with good/poor Stability Index, Odds Ratio, Mean ± SD, and p values for each sway direction pre-test and post-test.

**Pre-test (post-test) Sway Index (numbers injured/uninjured)**						
	**Anterior/posterior**		**Medial lateral**		**Overall**	
	**Injured**	**Uninjured**	**Injured**	**Uninjured**	**Injured**	**Uninjured **
**Number Good**	10(13)	5(2)	56(51)	11(11)	15(18)	5(2)
**Number poor**	54 (51)	8(11)	8(13)	2(2)	49 (46)	8 (11)
**OR**	3.38 (0.71)		0.79(1.40)		0.71 (0.47)	
**Pre-test Sway Index (degrees) **						
**Mean ± SD**	0.80 ± 0.40	0.69±0.26	0. 32±0.20	0.34±0.35	0.77 ± 0.43	0.70±0.32
**p (injured vs uninjured)**	0.35		0.78		0.58	
**Post-test Sway Index (degrees) **						
** Mean ±SD**	0.73 ± 0.35	0.99±0.49	0. 38 ±0.23	0.42±0.30	0.73 ± 0.37	0.98±0.51
**p(injured vs uninjured)**	0.03		0.59		0.04	

injured = 64, uninjured =13. Bold = good static balance.

**Table-II:** Numbers of injured and uninjured players with good and poor Limits of Stability (LOS) by direction Pre-test and Post-test, and Odds Ratios (OR).

		**Pre-test LOS**		**OR**	**Post-test LOS**		**OR**
**Directions**	**Group**	**Good**	**Poor**		**Good**	**Poor**	
**Forward**	injured	19	45	1.48	32	32	1.17
	uninjured	5	8		7	6	
**Backward**	injured	34	30	1.03	40	24	0.96
	uninjured	7	6		8	5	
**Right**	injured	33	31	1.10	43	21	1.10
	uninjured	7	6		9	4	
**Left**	injured	33	31	0.81	38	26	1.09
	uninjured	6	7		8	5	
**Forward Left**	injured	22	42	0.65	29	35	1.93
	uninjured	4	9		8	5	
**Backward Right**	injured	27	37	1.18	39	25	0.29
	uninjured	6	7		4	9	
**Backward left**	injured	30	34	1.81	37	27	0.85
	uninjured	8	5		7	6	

**Table-III:** Mean ± SD, and p values for LOS (%) directional control for each direction for injured and uninjured groups pre and post-test

	**Limits of stability (%) for each direction of movement**								
	**Front**	**Back**	**Right**	**Left**	**Front right**	**Front left**	**Back right**	**Back left**	**Overall**
	**Pre-test Mean ± SD**								
**Injured** **Uninjured**	54±16 62±21	68±19 70±17	65±15 70±19	68±15 70±14	60±14 65±15	58±14 64±16	62±15 67±16	64±14 67±17	56±12 61±13
**p**	0.12	0.73	0.30	0.66	0.25	0.17	0.28	0.50	0.18
	**Post-Test Mean ± SD**								
**Injured** **Uninjured**	54±25 68±19	68±19 70±16	64±14 72±12	68±17 71±15	51±14 60±16	59±13 64±12	65±11 62±17	65±15 61±18	55±15 60±13
**p**	0.06	0.72	0.05	0.06	0.04	0.20	0.42	0.40	0.27

Our findings of more moderate injuries is similar to that reported by Haseler et al. that 17 year old rugby players suffered more moderately severe injuries.^9^ These investigators attributed this to high school students deemphasizing the rehabilitation of their injuries.

Risk for injury in rugby varies by player position.^10^^,^^14^ Players occupying the scrum-half sustained the most injuries (14.8%) which was unsupported in the literature. The number eight position (8.2%) and loose-head prop (8.2%) in the forwards also sustained significantly more injuries as reported in other studies.^10^^,^^14^ Players in the number eight were commonly injured.^5^^,^^12^ Ripani et al. believed that the demands of rugby being faster with more attacking and collisions could attribute to higher risk for injury by the number eight players.^2^ Brooks and Kemp reported that tackling and scrimmaging by the loose-head prop could account for more injuries in the neck region in players in this position.^6^ The scrum-half reported more injuries in the lumbar spine sustained from passing the ball. In this study, the majority of injuries occurred in contact or collision, during matches (66%), particularly in the 2nd half (57%) similar to that reported by Barthgate et al.^12^

The findings related to static balance (SI) are related to the directional play required in rugby. Players joining a ruck or a maul participate from behind the team-mate and not from the opponent’s side, otherwise a penalty kick will be given. The increase in medial/lateral SI from pre-test to post-test resonates with de Freitas et al.^15^, who reported that medial/lateral sway decreases with strong knees, hips and trunk suggesting that improved joint stability will decrease SI.^15^ They also reported a decrease in anterior/posterior sway when the knees, hips and trunk were immobilized suggesting that improved stability of lower limb joints decreased sway. 

The mean pre- and post-season anterior/posterior SI was high suggesting poor static balance in this plane but the medial/lateral SI indicated acceptable static balance. The reduced static balance in the sagittal plane and overall, was similar to findings by Arnold and Schmitz.^16^ Their study assessed the relationship between the overall, anterior/posterior and medial/lateral sway index. Arnold and Schmitz used a Biodex stability system which has resistance settings not found in the Biosway balance system.^16^ In addition their tests were done in single leg standing and assessed rotation of axial movement rather than just postural sway. The differences in stability improvement in the two planes could be related to anatomical and biomechanical factors. Biomechanical variables included increased rotation in an anterior/posterior direction, and increased muscular stability in medial/lateral direction. The anatomical factors included the fact that the ankle’s range of motion in the anterior/posterior direction is greater than that in the medial/lateral direction. Hrysomallis et al. showed that ankle injuries were related to increased M/L sway.^3^ In the current study the wide range in SI scores may be related to the high incidence of ankle injuries. The slight decrease in mean M/L SI may be due to outliers. 

The overall and specific directional LoS increased post-test suggesting an improved dynamic balance. Postural control during goal-directed activities is important. Rugby players need to focus their attention on the specific tasks during play to maintain balance needed for the dynamic activities.^4^ A significant inverse correlation (R = -.218; p<0.05) between injury incidence and dynamic balance LOS forward right in the pre-test relates to the significant difference in directional control to the front and to the right between injured and uninjured players. There is no literature to which this finding can be compared. 

## CONCLUSION

Injury incidence in the selected cohort was lower than that reported in the literature. Injury incidence correlated with dynamic balance in the forward right direction. Dynamic balance to the right and forward was significantly different from that in uninjured players. Larger controlled studies are recommended.

## Notes

***Clinical relevance:*** The results of this study could be used to inform injury prevention and conditioning programmes for rugby players. Such programmes should include task specific and customised stability and balance training activities.

## Notes

***Conflicts of Interest and sources of funding*** *:* Jaco Ras received partial funding from the University of KwaZulu Natal to conduct this study as part of his master’s degree work.
